# DNA damage tolerance by recombination: Molecular pathways and DNA structures

**DOI:** 10.1016/j.dnarep.2016.05.008

**Published:** 2016-08

**Authors:** Dana Branzei, Barnabas Szakal

**Affiliations:** IFOM, The FIRC Institute of Molecular Oncology, Via Adamello 16, 20139 Milan, Italy

**Keywords:** DDT, DNA damage tolerance, DDR, DNA damage response, ss, single stranded, DSBs, Double strand breaks, TLS, Translesion Synthesis, PRR, Postreplication repair, HR, Homologous recombination, PCNA, proliferating cell nuclear antigen, SLDs, SUMO-like domains, STR, Sgs1-Top3-Rmi1, HJ, Holliday Junction, CFS, Common Fragile Sites, NPS, Natural Pausing Sites, Chromosome replication, DNA damage tolerance, Replication stress, Homologous recombination, Fork reversal, PCNA, Ubiquitin/SUMO modifications

## Abstract

Replication perturbations activate DNA damage tolerance (DDT) pathways, which are crucial to promote replication completion and to prevent fork breakage, a leading cause of genome instability. One mode of DDT uses translesion synthesis polymerases, which however can also introduce mutations. The other DDT mode involves recombination-mediated mechanisms, which are generally accurate. DDT occurs prevalently postreplicatively, but in certain situations homologous recombination is needed to restart forks. Fork reversal can function to stabilize stalled forks, but may also promote error-prone outcome when used for fork restart. Recent years have witnessed important advances in our understanding of the mechanisms and DNA structures that mediate recombination-mediated damage-bypass and highlighted principles that regulate DDT pathway choice locally and temporally. In this review we summarize the current knowledge and paradoxes on recombination-mediated DDT pathways and their workings, discuss how the intermediate DNA structures may influence genome integrity, and outline key open questions for future research.

## 1 Introduction

Accurate genomic duplication is essential for genome integrity, normal development and disease prevention [1]. This tremendous undertaking is made possible by a task-force of highly conserved replication and DNA repair factors that generally work with astonishing rapidity and accuracy. Part of this success is attributed to intricate regulation of DNA replication and metabolism factors in response to replication stress, which is extremely prevalent. Replication stress comes in different flavors and generally associates with DNA damage or DNA structures that impede replication [1]. In response to replication stress, single stranded (ss) DNA is often exposed proximal to replication forks, leading to local activation of DNA damage response (DDR) pathways. DDR promotes faithful completion of replication by cooperating with and regulating DNA metabolism factors to ensure recognition, bypass and repair of lesions [1], [2].

An important strategy to deal with replication-stalling lesions or DNA structures is to use specialized bypass mechanisms known as DNA damage tolerance (DDT) to replicate across the obstructing element, before attempting excision repair. Notably, if excision repair were to happen when the impediment for the replicative polymerase is encountered, while being present on ssDNA (the duplex DNA would have been already unwound by the replicative helicase), a double strand break (DSB) would be formed proximal to the fork. DSBs are extremely dangerous as their inappropriate repair is a leading cause for chromosomal rearrangements [3], [4]. Notably, as ssDNA is fragile, persistent ssDNA may cause breakage in the discontinuity region [5]. Thus, an important function of DDT is to prevent replication-associated DSBs *via* its role in mediating replication bypass across lesions, which in turn serves also the important scope of completing replication [2], [6].

Here we will summarize basic concepts of DDT, with a focus on recently emerged principles that govern the deployment, location and timing of DDT pathways, and discuss recent findings related to factors and structures that mediate damage bypass, which inform about the underlying mechanisms. We will highlight areas of future research and topics of debate, which will also bring into focus that DDT is at the nexus of various DNA metabolism pathways.

## 2 Two modes of DDT and three genetic ways for damage-bypass

Two modes or strategies of damage bypass or DNA damage tolerance (DDT) are highly conserved throughout the eukaryotic kingdom [1]. One mode involves usage of translesion synthesis (TLS) polymerases, which differently from replicative polymerases, can replicate directly across the lesions [7]. The trade-off for using TLS polymerases is increased risk to introducing mutations and, because of this, the TLS mode is considered to be error-prone. The other mode involves recombination to a homologous template, usually the sister chromatid, and is generally accurate in outcome [6].

**Fig. 1:**
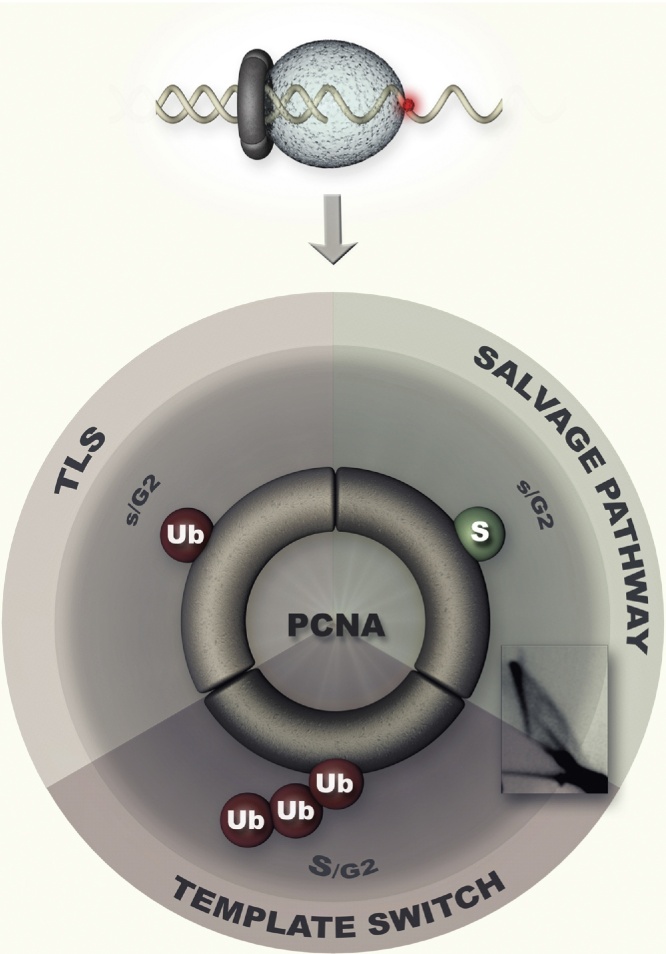
Stalling of the replicative polymerase, depicted as a ball in light grey, upon encountering of a lesion (depicted in red), triggers DNA damage tolerance (DDT) pathways. Translesion synthesis (TLS), Template switch and the Salvage pathway are the three main DDT pathways, and they are facilitated, mediated, or inhibited, respectively, by PCNA modifications with ubiquitin (Ub), polyubiquitin and SUMO (S). The cell cycle phases in which these pathways are preferred are also indicated. The recombination structures arising *via* template switch and the salvage pathways can be visualized by 2D gel analysis of replication intermediates, and their migration properties are identical.

Initially, DDT research revolved around genetic approaches that screened for mutations that impaired the ability of cells to tolerate exogenous damage. These endeavors revealed that a very important fraction of DDT is mediated by a so-called postreplication repair (PRR) pathway, in addition to the Rad51- and Rad52-mediated homologous recombination (HR) pathway (reviewed in Ref. [6]). The nomenclature of PRR was inspired by the crucial role of factors belonging to this pathway to promote filling of gaps left behind replication forks when cells were allowed to replicate in the presence of DNA damage [8]. PRR crucially depends on the conserved genes *RAD6* and *RAD18*, which encode for ubiquitin conjugating and ubiquitin ligase activities, respectively. TLS polymerases also belong to the *RAD6* pathway, but soon it became evident that the *RAD18* pathway contained activities different from TLS polymerases that were required to mediate damage bypass and gap filling *via* a recombination-like mechanism to the sister chromatid [9]. This recombination-like pathway, which was dependent on *RAD18-RAD6* genes, but genetically different from HR, was called template switching, although the mechanism and activities involved were puzzling. The Rad5 ubiquitin ligase and Mms2-Ubc13 ubiquitin-conjugating complex were later identified to belong to the template switch recombination branch of the *RAD18* pathway. Thus, three main genetic ways mediating DDT emerged: TLS, template switch, and HR, the latter also called the “salvage pathway” (Fig. 1 and see below).

Following this genetic categorization of DDT, efforts were made to understand how the choice between these pathways took place and on solving the puzzle as to how protein ubiquitylation mediated by the *RAD6-RAD18* pathway was important for lesion tolerance. A groundbreaking discovery in both these respects was that the polymerase clamp, PCNA (proliferating cell nuclear antigen), is mono- and polyubiquitylated by factors belonging to the *RAD6* pathway [10]. These modifications of PCNA are important for DDT and could potentially explain the distribution of labor between TLS and template switch in the context of the *RAD6-RAD18* pathway. Specifically, replication stress leads to exposure of ssDNA and recruitment of the ssDNA-binding protein, Rad18 [11], which together with Rad6, mediates monoubiquitylation of PCNA at a conserved residue, K164 [10]. The additional recruitment of Rad5 to ssDNA, and with it, of Mms2-Ubc13 [12], causes extension of monoubiquitylation to K63-linked polyubiquitin chains [10]. Monoubiquitylation of PCNA facilitates the TLS mode [13], [14], whereas polyubiquitylation mediates template switching, and inhibits the TLS mode [9], [15], [16], [17], [18] (Fig. 1). It is important to note, however, that PCNA functions as a trimer, and thus, more than one modification may occur on the same clamp (Fig. 1). Moreover, besides mono and polyubiquitylation, PCNA is also modified with SUMO at K164, and to a lesser extent at K127 in budding yeast [10], [19]. SUMOylation of PCNA allows template switching, but prevents HR (Fig. 1 and see below) [15], [16], [20]. The identified DDT pathways, key factors in these pathways, and PCNA modification with ubiquitin and SUMO are conserved also in higher eukaryotes, indicating that the DDT orchestration occurs by similar mechanisms also in vertebrates.

As PCNA modifications can both mediate and block specific DDT pathways (for instance PCNA polyubiquitylation mediates template switching, but counteracts TLS [17]), several important research areas for understanding DDT regulation will be to explore whether individual PCNA trimers carry more than one of these modifications *in vivo*, how dynamic these modifications are, if preference for an initial type of PCNA modification is induced by distinct replication stress cues, and whether the initial modification counteracts or rather supports subsequent modifications of the clamp with other types of ubiquitin or SUMO moieties. In other words, does the first modification on PCNA function to “lock” an individual clamp to serve in a specific DDT module (for instance, template switch or TLS), or do these modifications often cooperate or act sequentially to each other to allow fluidity and cooperation between different DDT pathways, as in the case of complex lesions?

A conundrum related to PCNA modifications and their roles in mediating specific DDT pathways comes out from recent observations that pinpoint cooperation between factors mediating PCNA polyubiquitylation and HR in the context of template switching ([20] and see Section 3). As HR is inhibited by PCNA SUMOylation [15], [16], how PCNA modifications with polyubiquitin and SUMO relate to each other to allow template switch is currently not understood. Recent work, however, revealed that DDT regulation at damaged sites is significantly more complex than just the one emerging from considering the effects of PCNA modifications, and additional factors are recruited to regulate the local effect of PCNA modifications. SUMOylated PCNA, a PCNA modification induced during replication [10], suppresses HR at stalled forks [15], [16], *via* its ability to recruit the anti-recombinase Srs2, a helicase that disrupts Rad51 filaments [21], [22]. However, local assembly of Rad51 at damaged sites is facilitated by the conserved SUMO-like domains (SLDs)-containing factor, Esc2, which binds to replication-associated DNA structures and to SUMOylated PCNA readers, Elg1 and Srs2, facilitating local down-regulation and turnover of Srs2 [23], [24].

In addition to PCNA modifications with ubiquitin and SUMO, regulatory pathways involving chromatin structure and genome architecture also emerged in recent years as important players in mediating preference of one DDT pathway versus another, both locally (at a damaged site) and temporally (in the context of cell cycle and replication timing) [25], [26], [27], [28]. Very little is currently understood about this larger picture in which DDT takes place and the mutual relationship envisaged to exist between DDT and chromatin. Moreover, in spite of our understanding of genetic pathways and factors contributing to different DDT modes, mechanistic questions as to how recombination-mediated DDT is similar to or differs from canonical HR, and methodologies to visualize and identify the DNA structures implicated in these reactions, only recently began to evolve.

## 3 Commonality and divergence between template switch and the salvage pathway

Although template switch is a recombination process, as revealed from examining the final outcome of the bypass reaction [29], it is genetically distinct from the one of HR, as assessed by classical epistatic studies. The question as to whether template switch is manifested through recombination structures (the PRR pathway was initially known as non-recombination gap filling mode [8]) remained a puzzle until 2D gel analysis of DNA replication intermediates revealed a population of recombination-like structures consisting of sister chromatid junctions (SCJs) that had the genetic dependencies of template switch intermediates [20] (Fig. 1). These molecules are induced proximal to replication fork structures when replication is challenged by DNA damage and accumulate in mutants defective for the conserved RecQ helicase Sgs1 [20] (BLM in mammalian cells), already reported to function together with the topoisomerase Top3 to resolve recombination intermediates *in vivo* and *in vitro* [30], [31]. The DDT defects of *sgs1* are also epistatic with the ones caused by mutations in factors belonging to the error-free branch of the Rad18 pathway [20], [32], altogether indicating that Sgs1 functions downstream of PCNA polyubiquitylation in the error-free recombination-mediated DDT pathway by resolving or remodeling template switch intermediates (see also below). Interestingly, however, the formation of Rad18- and PCNA-polyubiquitylation-dependent SCJs require also Rad51 and recombination mediator activities, leading to the concept that PRR and HR activities cooperate during template switching [20], [33].

However, in backgrounds defective in PCNA SUMOylation, recombination structures involving SCJs that are independent of Rad18 and PCNA polyubiquitylation activities, but have identical migration properties in 2D gel electrophoresis, mediate DDT [20] (Fig. 1). These molecules still require HR activities for formation and depend on Sgs1 for resolution [20]. Later studies uncovered that this HR-dependent but Rad18-independent DDT recombination pathway is preferentially deployed later in cell cycle in comparison with template switching, which preferentially occurs early in S phase [26], [34], [35]. The PCNA polyubiquitylation-independent recombination-mediated DDT pathway is often called the “salvage pathway”, as it is thought to deal with lesions that escaped the time window of template switching, although how this window is defined remains still poorly understood (see also Section 4).

Because current 2D gel combined with genetic approaches allow the distinction of the recombination structures arising *via* template switch versus the salvage pathway proximal to early origins of replication, our knowledge on DDT should improve in the next years to reveal factors that contribute to one or both pathways of recombination-mediated DDT, unveil their interplay with PCNA modifications, and allow deductions on their temporal preference of action. Already in regard to template switching, the repertoire of factors contributing to the formation or safeguarding the stability of the intermediates extended significantly, revealing for instance that template switching is prevalently initiated at DNA gaps enabled by replicative helicase-coupled repriming, that the strand invasion process is facilitated by gap extension, and that DNA polymerase delta plays a major role in the DNA synthesis step of this reaction [20], [25], [34], [36].

**Fig. 2:**
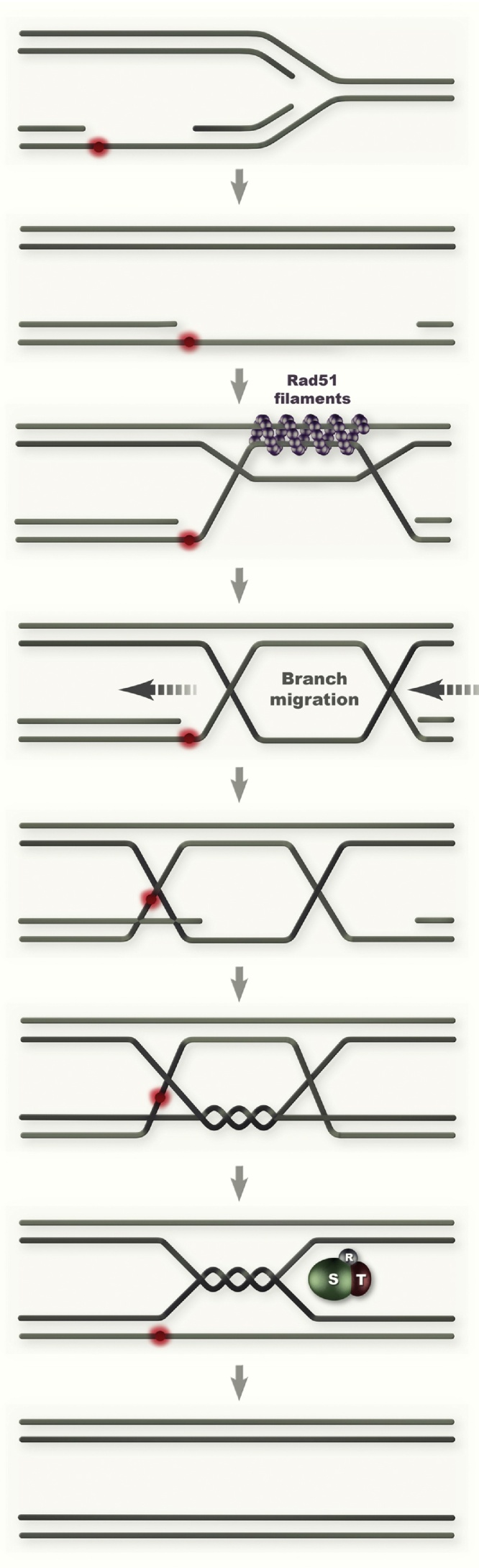
Schematic representation of postreplicative gapfilling by template switching. Here, the DNA lesion is shown in red on the leading strand reactivated by repriming downstream of the lesion. See text for details. STR stands for Sgs1-Top3-Rmi1.

More recently, template switch intermediates arising proximal to an origin of replication following replication stress by methylating damage, were visualized by electron microscopy (EM) [37]. This approach brought evidence for the postreplicative model of template switching (postreplicative gaps priming strand invasion into the homologous duplex) and revealed the nature of key intermediates implicated in this reaction (see below). The emerging model of template switching illustrated in Fig. 2 is that the gap region anneals to the homologous duplex, causing the formation of a three-stranded DNA structure before the newly synthesized strand from the undamaged duplex starts to be used as template for synthesis. This causes the formation of a D-loop structure that is subsequently extended to a double Holliday Junction (HJ)-like intermediate (pseudo-double HJ), in which the newly synthesized strands are paired by plectonemic pairing induced by DNA synthesis, and the parental strands are annealed to each other (Fig. 2). The pseudo-double HJ intermediates are then dissolved to hemicatenanes by the Sgs1-Top3-Rmi1 complex, before full resolution takes place, likely by Top3 (Fig. 2). Moreover, parallel analysis of EM intermediates in wild-type and *sgs1* revealed the same types of intermediates in both backgrounds, but with a relative enrichment in structural intermediates requiring Sgs1 action for resolution [37]. These results thus bring validation that the employed 2D gel approach to screen factors contributing to recombination DDT intermediate formation/stability in an *sgs1* background is suitable to uncover activities that physiologically contribute to this process. Notably, during this recombination process of template switching, various structures may become targeted by nucleases, in which case deleterious outcomes in terms of crossovers are expected (Fig. 2). In this vein, recently it was revealed that Mus81-Mms4 nuclease acts on such intermediates, but its action is prevented until G2/M by the replication checkpoint, which in this way safeguards error-free formation and resolution of DDT recombination intermediates [38]. These results expose thus a function of the checkpoint in the context of postreplicative DDT, which is different from its role at stabilizing stalled replication forks (see below).

To what extent the salvage pathway is different from template switching in terms of contributing factors, the architecture of the DNA intermediates involved, and accuracy of the outcome remains largely unknown. If the salvage pathway represents the minimal set of a HR rescue team operating from S to G2/M to which additional factors are added earlier in S phase by means of enzymatic activities that induce or are coincident with PCNA polyubiquitylation is currently elusive. Is PCNA polyubiquitylation making the recombination reaction of gap filling more accurate? The reason of why PCNA poyubiquitylation itself is required for template switching is rather unclear. Are the polyubiquitin chains on PCNA functioning as a sort of glue that may locally concentrate factors required to mediate or facilitate this recombination reaction or the proximity to the sister chromatid? What would be those additional factors required for template switch-mediated DDT, but not for the salvage pathway? Which are the factors and intermediates common to template switch and the salvage pathway? And finally, is there a rationale in terms of genome integrity in preferring template switching in S phase or in postponing the salvage pathway to later times in the cell cycle? Does PCNA polyubiquitylation ensure that this recombination process takes place preferentially behind replication forks rather than at forks where HR-mediated rescue is generally error-prone [39]? With many open questions to be explored lying ahead, the coming years should witness an important broadening in our knowledge on DDT mechanisms and their regulations.

## 4 Timing and location of DDT pathways and their cooperation with fork stabilization mechanisms

**Fig. 3:**
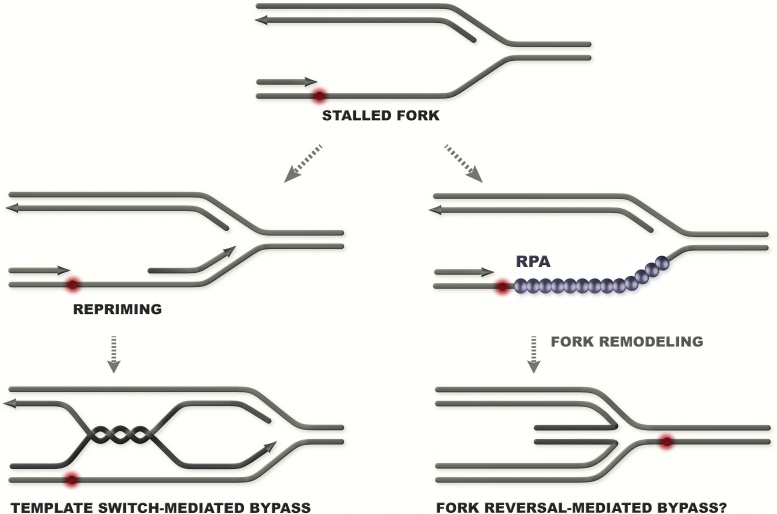
Schematic representation of two main events that promote fork stabilization and damage bypass upon stalling of the leading polymerase. Repriming is a crucial step for postreplicative DDT by template switch (represented) or TLS. Fork reversal is mediated by various fork remodeling activities *in vitro* and can promote in certain conditions replication fork stabilization and DDT.

When the replicative polymerase encounters a blocking lesion or DNA structure, a transient stalling will occur. If the damage is placed on the lagging strand, synthesis of a new Okazaki fragment will soon cause the obstructing damage to be contained in a small DNA gap behind the replication fork. In such circumstances, the lesion bypass will take place in the context of post-replicative gap filling using either TLS or recombination modes (Fig. 1). Stalling on the leading strand, however, can potentially cause uncoupling of the leading and lagging strand polymerases, leading to the formation of a ssDNA stretch close to the fork junction, if repriming is not initiated downstream the lesion (Fig. 3). This is problematic because ssDNA is fragile and such forks, or the ssDNA within them, should be at least protected until a converging fork can complete replication in the region. In certain conditions, however, the probability of converging forks rescuing the halted replication fork are low, as is the case of origin-poor regions, regions of unidirectional replication or regions that contain a large number of natural obstacles for fork progression, such as ribosomal DNA (rDNA) and vertebrate centromeric DNA. The forks in such problematic regions will likely need to be ultimately rescued by HR-mediated fork reactivation mechanisms before or after a DSB occurred, that is, by DSB-dependent and independent HR mechanisms (reviewed in Refs. [39], [40]). However, recent evidence suggests that such HR-dependent fork reactivation mechanisms are liable to generate genetic instability by instigating microhomology-driven strand invasion or by establishing a replication fork whose progression is error-prone [39]. Due to their error-proneness, it will be desirable for genome integrity that HR-mediated fork rescue mechanisms are postponed as last-resort options. If and how this is achieved is not well understood, but perhaps the observed temporal preference of recombination-dependent DDT pathways (Fig. 1) is facilitated by fork stabilization mechanisms. In this vein, we propose that the observed delayed timing of the HR-dependent salvage pathway reflects its potential toxicity in terms of outcome. The increased preference for its use in G2/M might not therefore primarily reflect its post-replicative nature at DNA gaps that could not be filled in by template switching (Fig. 2), but also its action at regions where repriming followed by PRR is intrinsically difficult to achieve, such as at regions where replication fork progression is blocked. This latter scenario is different from experimental situations in which genetic mutations in factors affecting PCNA polyubiquitylation and or SUMOylation cause anticipated reliance on HR factors without the contribution of PCNA polyubiquitylation [20]. These latter conditions, unless coupled with problematic genomic regions or difficult to surpass replication fork barriers, may only instigate postreplicative gapfilling by HR without the help of PCNA polyubiquitylation. Postreplicative Rad18-indepent HR may not necessarily cause significant differences in the DNA intermediates being formed, nor be associated with increased error-proneness of the DNA synthesis implicated in gap filling. Nevertheless, these effects are interesting to investigate as such research has the potential to reveal the role and purpose of PCNA polyubiquitylation in template switch. However, it is crucial to bear in mind that both the genomic context and the availability of factors to be used for recombination-mediated DDT are important in determining the outcome. Here we start by reviewing evidence for postreplicative DDT and then discuss on mechanisms of fork stabilization that may also be implicated in DDT following specific types of replication stress.

### 4.1 Evidence for post-replicative gap-filling DDT

Yeast and mammalian cells replicating in the presence of DNA damage accumulate gaps behind replication forks, and these gaps are subsequently filled in a manner that depends on DDT activities (reviewed in Ref. [41]). EM analysis of replication intermediates brought evidence for the formation of gaps on both strands as well as for the intermediates implicated in gap-filling [37], [42]. These observations are consistent with the fact that limiting critical error-free DDT components and TLS factors to G2/M allows normal tolerance of lesions [32], [43], and bring strong support for the postreplicative DDT as the prevalent mechanism.

The postreplicative model of DDT envisages that blocked DNA synthesis on either leading and lagging strands would often recommence *via* repriming (Fig. 3). Such repriming downstream the lesion can be mediated in principle by the Primase activity of Polα/Primase, or by specialized polymerases with Primase activity, such as Prim-Pol in human cells [44], [45]. *In vivo* results in budding yeast indicate a crucial role for the Polα/Primase activity in recombination-mediated DDT [25]. Interestingly, these repriming events appear to be generally coupled to replicative helicase movement as mutations in factors that are not required for the Primase activity, but architecturally bridge the replicative helicase, MCM, with the Polα/Primase activity also cause severe defects in recombination-mediated DDT, and in postreplicative template switching [25]. Thus, in response to transient stalling, repriming on the leading strand happens in coordination with fork movement and perhaps in coordination with new Okazaki-fragment synthesis on the lagging strand. When such repriming is impaired, an increase in reversed fork structures is observed [25] (Fig. 3). But whether the reversed forks observed in Primase mutants provide means for alternative DDT or only serve to stabilize the fork remains still unknown. Primase mutants, however, rely on Rad52/Rad59 annealing activities for survival and show elevated increase in faulty-annealing events [25], suggesting that reversed forks in this context may instigate genome instability if used as alternate DDT. Moreover, as many enzymes can catalyze fork regression on naked substrates, including enzymes associated with DDT recombination function, such as Rad5, HLTF (one of the Rad5 orthologs in human cells), FANCM/Mph1 [46], [47], [48], [49], it is likely that in certain circumstances, fork regression mediates recombination-mediated DDT. Uncovering those contexts *in vivo* and deciphering the effects of fork reversal-initiated DDT events on genome instability would be challenging, but important for our understanding of DDT.

The fact that repriming is an integral part of DDT initiated by both leading and lagging strand template lesions is also evidenced by the fact that factors that contribute to gap processing, such as 9-1-1 and Exo1, strongly affect template switching, beyond what it were expected if their contribution were limited to processing of Okazaki fragments (lagging strand gaps) [34], [36]. Thus, genetic, molecular and structural data are consistent with postreplicative template switching as a prevalent DDT mechanism in conditions when repriming is proficient.

### 4.2 Fork stabilization mechanisms and their role as regulators of DDT

Certain replication stress contexts that perturb replication are not amenable to repriming. These are for instance lesions, such as interstrand crosslinks, that block the progression of the replicative helicase, or regions containing multiple intrinsic replication obstacles, such as DNA-bound proteins, highly transcribed genes or heterochromatin with repressed genes. Such DNA lesions and chromatin organization patterns block fork progression and greatly increase the chances of fork fragility. Rescue by HR in those contexts has often proved to be error-prone and instigate homology-driven genome instability [39], [40]. In those cases, fork stabilization, rather than DDT, is best suited as the primary line of defense.

**Fig. 4:**
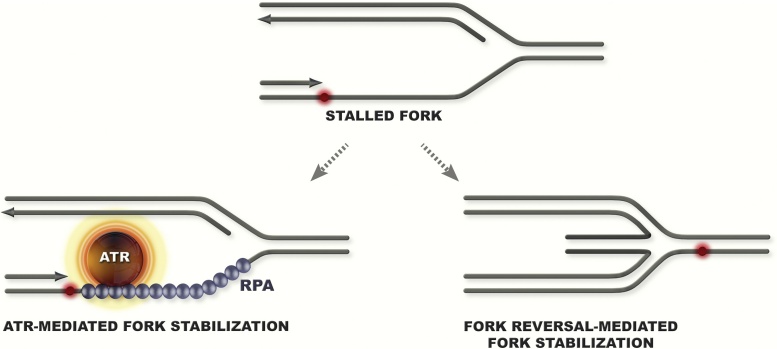
Schematic representation of fork stabilization mechanisms mediated by the replication checkpoint ATR and fork reversal.

One well-studied mechanism of replication fork stabilization is the one provided by the replication checkpoint (Fig. 4). The checkpoint is activated by the RPA-bound ssDNA, and regulates several important aspects of the replication program to prevent fork-collapse. These functions include regulation of origin firing to prevent RPA exhaustion, stabilization of the replisome, and regulation of enzymes with replication fork processing activity to prevent fork collapse (reviewed in Refs. [1], [50]).

A paradigm for chromosomal loci predisposed to fragility are common fragile sites (CFSs) and regions containing natural pausing site (NPS) elements. The replication checkpoint is crucial for preventing CFS breakage [51], [52], although to what extent this function of the checkpoint is local or aids in completing genome duplication to prevent breakage of CFSs in mitosis remains unknown [53], [54]. Recently, the Structural maintenance of chromosomes (SMC5/6) complex was shown to localize and prevent fragility of NPSs in budding yeast [55], [56] and to localize to early fragile sites in mammalian cells [57]. NPSs are regions that resemble CFSs and are hot-spots for replication-associated fragility in budding yeast [58]. The mechanism involved is likely different from the one of checkpoint, as *smc5/6* mutants are capable to boost checkpoint activation, and likely relates to local regulation of HR activities. In *smc5/6* mutants, recombination structures that are instigated by prolonged replication fork pausing and genetically depend on the conserved Tof1/Csm3 (Tim-Tipin) replication fork-pausing complex accumulate at NPSs [55]. It is likely that those unrestrained HR events underlie fragility, but whether the function of Smc5/6 in this context is topological, manifested by its ability to accommodate the topological changes associated with prolonged pausing as to prevent HR initiation, or rather enzymatic, manifested by its ability to disrupt recombination intermediates once they are assembled to prevent their maturation remains unknown. One proposed role for Smc5/6 in relation to recombination regulation, based on *in vitro*-activities, is to prevent Mph1-mediated fork regression [48], an event that can, in principle, instigate recombination events by promoting strand invasion into the parental duplex ahead of the stalled fork to promote fork restart.

However, besides the potential roles of reversed forks to mediate (toxic) DDT (Fig. 3), recent work provides evidence that fork regression often functions as a fork stabilization mechanism, especially in mammalian cells [59]. While fork reversal is increased upon treatment with various DNA damaging agents, and is mediated by RAD51 [60], whether it represents a means to promote DDT or simply functions to stabilize stalled forks remains yet to be elucidated. Considering the increased occurrence in problematic regions in mammalian cells, we favor the idea that fork reversal would primarily serve to transiently stabilize stalled forks. We also propose that RAD51-mediated fork stabilization and fork-regression pathway may act in parallel or in compensation with the one provided by the replication checkpoint (Fig. 4), or may be preferentially used at stalled replication forks where the replisome already disintegrated. This may explain why budding yeast replication checkpoint mutants show increased fork reversal upon conditions that induce fork stalling [61] and have increased reliance on Rad52 activities [62], which in budding yeast may act preferentially over Rad51 to induce fork reversal [25], [63]. Our view is thus that regressed forks provide a means to stabilize stalled forks and buy time until a converging fork can merge with the arrested one. However, as reversed forks can be targeted by processing enzymes and initiate error-prone recombination [64], it is likely that in contrast to checkpoint activation, their formation is not globally acclaimed, but rather locally induced and controlled *via* mechanisms that still require elucidation.

## 5 Summary

DDT mechanisms are crucial to prevent fork breakage and to promote replication completion, by promoting damage bypass DNA synthesis either at the fork or postreplicatively. The temporal coordination of DDT pathways that ensure preferential usage early during replication to error-free postreplicative template switching is still not well understood, but likely involves regulation of replication factors by posttranslational modifications and fork stabilization mechanisms triggered by local chromatin and genomic features that block replication fork progression. Understanding how these DDT pathways are deployed and interrelate to each other is crucial for our knowledge of genome integrity mechanisms and replication stress responses. Moreover, from a practical point of view, as cells treated with chemotherapeutic agents crucially depend on DDT for their proliferation, understanding DDT mechanisms, their preferential deployment in different replication stress contexts and their impact on chromosome structure may inform therapeutic approaches by providing targeted inhibition. While many challenging questions remain, improved tools, approaches and working models hold promise to bring insight into the mechanism and intermediate products through which recombination-mediated DDT affects genome integrity.

## Conflict of interest

The authors declare no conflict of interest.
